# Glycerine

**Published:** 1878-02

**Authors:** 


GLYCERINE,

As an article of food, as a nutrient, is well worthy of being brought into public notice. Sweet oil in Palestine and other old countries, has for ages been used as an article of daily food, and glycerine may be considered as the essence of pure "sweet", that is "olive oil," they being one and the same; it is a perfectly neutral and bland fluid, and the most penetrating perhaps in all nature. Oil itself will permeate where water will not; and glycerine, which may be considered the ethereal part of oil, has this property to a most remarkable degree; it penetrates the solid bone; if poured into a mixture of blood and matter, such as is expectorated from consumptive lungs, it will get in between the globules of each and show them with great distinctness; being thus penetrating, it is the very best application for all feverish sores, for inflamed or dry surfaces simply from its quality of penetration and want of evaporatability; the first and highest value of any poultice is its capability of keeping moist for the longest time; no one ever thinks of a dry poultice; glycerine keeps a part moist longer than any substance known, hence its value as above, mixed with an innoxious dry powder, called sub-nitrate of Bismuth, so as to make a thin paste or poultice. It is one of the very best applications known for

BURNS, whether in children or adults, giving an almost instantaneous relief from suffering, by its entire exclusion of the air and by its moistening, hence cooling, soothing effects, promotes a speedy healing process, always safe, simple and efficient. A few cents will buy half a pound of it at any good drug store, and every family should have some at hand, in a bottle, plainly labelled, with a bottle of glycerine at its side.

If glycerine alone is applied with a common brush to the surface of the throat in that dangerous malady dyptheria, in a few minutes its permeative quality enables it to sink into and between the molecules of the false membrane, dissolving and than detaching it, in a few hours; so also in that other most alarming malady, the croup of little children, as it not only penetrates and thus disintegrates, spreads apart the tissues of the false membrane, but when it gets to the bottom of it, it spreads out between the layer of the membrane and the more natural mucous surfaces beneath and thus not only detaches the membrane, but imparts a healing, soothing influence to the inflamed condition of the natural surfaces. Hence also if applied to

DRY SORES, its affinity for organic globules is such that it seems to be attracted to them through the dry scab, moistens it, detaches it, and causes a new and healthful surface to grow beneath it; it has the same delightful, soothing and healing effects when applied to

BLISTERED SKIN, taking out the inflammation, cooling the parts and restoring them to their natural condition.

PAINFUL SORES

are very gratefully and speedily relieved by a mixture of morphine and glycerine. But more striking benefits are observed in its use as food, from its inherent nutritious qualities in all that large class of cases where a person needs

FOOD RATHER THAN PHYSIC.

As evidence of its capacity to fatten children a physician states in the American Journal of Pharmacy, its chemical formula being six parts carbon, seven parts hydrogen and five parts oxygen,

1st. An infant six months old, recovering from a severe diarrhœa, kept quite emanciated and pale. Glycerine was ordered for it, and in a few days a change was remarked in its appearance for the better, and in four weeks it weighed eight pounds heavier. 2. A child, sixteen months old, had its head covered with one continous scab,—porrigo. This was a family complaint, and resisted all manner of treatment for a very long time in all the other children. In the above case I resorted to glycerine, both internally and externally.

A cure was effected in three months. 3. A girl, seven years old, recovering from measles, retained her cough, emaciation, and nervous irritability. Dullness over apex of left lung; roughened breathing. No doubt the case was chronic pneumonia. Glycerine, as a last resort, was ordered in teaspoonful doses in water, three times a day. Recovery in six weeks. 4. A strumous boy, much emaciated, had hacking cough and night sweats. Pulse frequent. Sleep disturbed. Abdomen tumid and enlarged. Cervical glands swollen. Bowels irregular. Fecal discharges clay-colored. His case was such, that no one expected any more than a partial palliation. After other treatments had failed, I ordered glycerine in teaspoonful doses, in which were dissolved four grains of ferri ammon. cit., and one half of a grain of quinia, four times a day. This he continued for a year, and was in remarkably good health three years after.

